# Carbapenemase-Producing Enterobacteriaceae (CPE) Newborn Colonization in a Portuguese Neonatal Intensive Care Unit (NICU): Epidemiology and Infection Prevention and Control Measures

**DOI:** 10.3390/idr13020039

**Published:** 2021-05-01

**Authors:** Teresa L. Almeida, Tânia Mendo, Raquel Costa, Cristina Novais, Mónica Marçal, Filomena Martins, Madalena Tuna

**Affiliations:** 1 Neonatal Intensive Care Unit, Centro Hospitalar Lisboa Ocidental, Department of Pediatrics, Hospital de São Francisco Xavier, 1449-005 Lisboa, Portugal; mendotania.tfdsm@gmail.com (T.M.); raquelfreitascost@gmail.com (R.C.); criscnovais@gmail.com (C.N.); mlcarvalho@chlo.min-saude.pt (M.M.); mtuna@chlo.min-saude.pt (M.T.); 2 Department of Pediatrics, Hospital do Espírito Santo de Évora, EPE, 7000-811 Évora, Portugal; 3 Department of Pediatrics, Hospital José Joaquim Fernandes, ULSBA, 7801-849 Beja, Portugal; 4 Paediatric and Neonatal Functional Unit, Department of Paediatrics, Hospital de Cascais, 2755-009 Alcabideche, Portugal; 5 GCL-PPCIRA, Grupo Coordenador Local de Prevenção e Controlo da Infecção e Resistência aos Antimicrobianos, Centro Hospitalar Lisboa Ocidental, 1449-005 Lisboa, Portugal; fjmartins@chlo.min-saude.pt

**Keywords:** multidrug-resistant gram-negative bacteria, carbapenemase-producing Enterobacterales, neonatal intensive care unit, colonization, newborn

## Abstract

Infections due to carbapenemase-producing Enterobacterales (CPE) are increasing worldwide and are especially concerning in a neonatal intensive care unit (NICU). Risk factors for CPE gut colonization in neonates need to be clarified. In this work, we describe the epidemiological and clinical features of CPE-colonized newborns and the infection control measures in a Portuguese NICU. We performed a prospective, observational, longitudinal, cohort study for surveillance of CPE colonization. Maternal and neonatal features of colonized newborns and surveillance strategy were described. A statistical analysis was performed with SPSS23.0, and significance was indicated by *p*-value ≤ 0.05. Between March and November 2019, CPE was isolated in 5.8% of 173 admitted neonates. Carbapenemase-producing *Klebsiella pneumoniae* were the most frequently isolated. There was no associated infection. Birth weight, gestational age, length of stay, and days of central line were the identified risk factors for CPE colonization (bivariate analysis with Student’s t-test or Mann–Whitney U test, according to normality). No independent risk factors for CPE colonization were identified in the logistic regression analysis. CPE colonization risk factors are still to be determined accurately in the neonatal population. Active surveillance and continuous infection control measures restrained the current cluster of colonized newborns and helped to prevent infection and future outbreaks.

## 1. Introduction

Infections due to antibiotic-resistant Gram-negative organisms are increasing worldwide and represent a significant public health issue [1,2,3]. Newborns in a neonatal intensive care unit (NICU) are especially vulnerable to colonization and infection by several pathogens, including multidrug-resistant (MDR) Gram-negative bacteria. These severe infections entail significant morbidity and mortality in an NICU [3,4,5,6,7].

Gram-negative bacteria have developed several resistance mechanisms, including extended-spectrum β-lactamases (ESBL), AmpC cephalosporinases, and carbapenemases [8]. Carbapenems are usually used as a last resort antibiotic therapy for severe infections caused by MDR Gram-negative bacteria, including ESBL and AmpC producing bacteria [1,8]. However, the recent increase of carbapenemase-producing Enterobacterales (CPE) has become a challenge in neonatologists’ daily practice due to limited therapeutic resources. The most common carbapenemase-producing Enterobacterales is *Klebsiella pneumoniae* followed by *Enterobacter* spp. [2,5,9].

Carbapenem resistance can occur by an enzymatic (carbapenemase production) or nonenzymatic mechanism (expression of AmpC and decreased membrane permeability to carbapenems). Carbapenemase genes are highly mobile, enabling a rapid and frequent transference of multiple other antibiotic resistance genes [2,8].

The most commonly used classification of carbapenemases is the Ambler classification, according to amino acid sequence: Classes A, B, and D. Class A carbapenemases are found in Enterobacterales, *Pseudomonas aeruginosa*, and *Acinetobacter* spp. and include enzymes that hydrolyze all β-lactams, monobactams, and carbapenems (penicillinases, ESBL TEM, SHV and CTX-M, and K. *pneumoniae* carbapenemase). Class B carbapenemases are metallo-β-lactamases (MBL), including VIM (Verona integron-encoded metallo-β-lactamase) and NDM (Delhi metallo-β-lactamase), which provide resistance to all β-lactams and carbapenems. Class D carbapenemases, oxacillin-hydrolyzing β-lactamases (OXAs), have several variants and can hydrolyze penicillin and meropenem, but not extended-spectrum cephalosporins and aztreonam [2,8].

Neonatal risk factors for intestinal CPE colonization and infection have been inconsistently described in the literature, and these are mainly derived from adult studies [1]. Features reported include low gestational age, low birth weight, intensive care unit stay, prolonged hospitalization, prior exposure to antibiotics (especially carbapenems), invasive medical procedures or devices, history of surgery, congenital/syndromic conditions leading to impaired functional status and/or immunosuppression, lack of breastfeeding, maternal-to-neonatal transmission, and travel from endemic regions [1,6,9,10,11].

NICU outbreaks may have serious neonatal implications, and thus maintaining a high level of suspicion is crucial for prompt identification and outbreak control [7,12]. An outbreak in an NICU should be suspected when there are at least two newborns colonized with the same species, a single case of a rare Gram-negative bacteria identification, or a single systemic infection with an extended-spectrum β-lactamase producing or carbapenem-resistant Gram-negative bacteria [11,13].

There is a significant lack of data on the epidemiology, risk factors, and outcomes of MDR Gram-negative bacteria in Portuguese NICU, and in particular CPE colonization/infection [13,14]. Therefore, this study aims to describe the epidemiological and clinical features of CPE-colonized newborns, as well as infection control measures applied to prevent cross-transmission.

## 2. Materials and Methods

We performed a prospective, observational, longitudinal, cohort study for the surveillance of CPE colonization in the admitted neonates from March to November of 2019 in a Portuguese NICU, in Lisbon. Our NICU has 14 beds and an average of 250 admissions per year, providing care for preterm and term neonates.

All newborns admitted to the NICU were screened for the presence of CPE. The screening was performed using rectal swabs that were collected at admission, between 24 to 48 h of hospital stay, followed by weekly rectal swabs during the hospitalization period. This methodology was used for first-time admissions and for readmitted patients who had to leave the NICU for medical or surgical procedures.

Contact isolation measures were applied to all newborns until negative results in the two initial swabs. Upon positive screening, contact isolation measures with patient cohorting and use of disposable gowns and gloves during contact with the newborn was maintained until hospital discharge.

Rectal swabs were collected by the NICU’s nurses using a standard procedure. Swabs were collected on a sterile Stuart medium. CPE identification was made by polymerase chain reaction. Swabs from all neonates were screened for CPE according to the Centers for Disease Control and Prevention (CDC) criteria [15].

Collected data included the following: total number of NICU’s admissions, number of CPE-colonized patients, date of birth, gestational age, birth weight, type of delivery, time of membrane rupture, amniotic fluid characteristics, maternal history (nationality and recent traveling to endemic regions, days of hospital stay before labor, antibiotics during pregnancy and prenatal routine screening), date of admission and discharge, hospital length of stay, microorganism identification, type of carbapenemase, antibiotic susceptibility, day of CPE colonization, number of infected patients, comorbidities, cytopenia, hemoderivative transfusions, previous exposure to antibiotics, invasive techniques (mechanical ventilation, central lines, nasogastric intubation, surgery), enteral and parenteral feeding, isolation measures, and mortality.

A CPE colonization was defined as a CPE-positive rectal swab in a newborn without evidence of signs or symptoms of infection. A CPE infection was defined as a positive CPE specimen from a normally sterile site (e.g., blood or cerebrospinal fluid) together with signs and symptoms of infection. Definition of an outbreak included two or more sterile site isolates of the same species, with the same antibiogram, from different patients, within a period of two weeks. Alert should be triggered when there is either a case of three or more babies colonized with the same Gram-negative bacteria (in NICUs that routinely screen for colonization), a single case of a rare or never seen Gram-negative bacteria, or a single systemic infection with an ESBL-producing or carbapenemase-producing Gram-negative bacteria [11,13]. Imported cases were defined as positive swabs within the first 48 h after readmission. Cross-transmission was assumed in patients with two initial negative rectal swabs and subsequently acquired CPE colonization.

Categorical variables were presented as frequencies and were compared with Fisher’s exact test or a Chi-square test. Continuous variables were expressed as means and standard deviation values or as medians and minimum and maximum values. A bivariate analysis for continuous variables was performed with Student’s t-test or the Mann–Whitney U test, according to normality. A binary logistic regression model was performed. Statistical significance was indicated by a p-value ≤ 0.05. Analysis was performed with the IBM Statistical Package for Social Science (SPSS) for Windows, version 23.0 (IBM Corp., Armonk, NY, USA).

## 3. Results

Between 1 March and 30 November 2019, 173 newborns were admitted to the NICU, and 465 rectal swabs were performed. During this period a total of 10 patients (5.8%) were colonized with CPE. The first CPE-colonized patient was identified on 30 May, and the last one on 8 November. Four neonates had KPC *K. pneumoniae* isolation (all in May), two had OXA-48 *K. pneumoniae* isolation (in August), one had VIM-producing *K. pneumoniae* (in September) and three had VIM-producing Enterobacter cloacae (in November). Maternal and newborn features of colonized neonates are described subsequently.

Nine mothers were Portuguese and one was Brazilian. One mother had a history of recent travel to Asia. Four babies were born from multiple pregnancies with one set of triplets. All mothers were hospitalized before labor with a median of five days (minimum 2 days; maximum 13 days). Prenatal routine surveillance was normal in all cases, and one mother received antibiotics (no carbapenem) during labor. Two babies were born by normal vaginal delivery; one was born by instrumental delivery, and seven were delivered by cesarean. All amniotic membranes ruptured less than 18 h before delivery with clear amniotic fluid, except for one that had meconium-stained amniotic fluid.

Nine newborns were preterm, all with a birth weight <2500 g, and two with extremely low birth weight (<1000 g). Median gestational age was 30 weeks (minimum 27 weeks; maximum 39 weeks), and median birth weight was 1596 g (minimum 502 g; maximum 3700 g). Eight neonates were male. Nine neonates were admitted to the NICU immediately from the delivery room. One newborn with a syndromic comorbidity underwent cardiac surgery and was admitted from another hospital. Another newborn left the NICU ward for a radiologic procedure and was readmitted afterward.

Prior to CPE identification, five newborns were exposed to intravenous antibiotics, and one required topical oxytetracycline for acute conjunctivitis. Antibiotics previously used were ampicillin (4 patients), gentamicin (4 patients), vancomycin (3 patients), amikacin (2 patients), and ceftazidime, piperacillin/tazobactam, and meropenem in one patient. In this group, the median number of antibiotics used per patient was two (minimum 1; maximum 7), and the median length of antibiotic therapy per 1000 patient-days was 12 days (minimum 8 days; maximum 99 days).

All newborns were exposed to invasive techniques. Specifically, 2 patients were submitted to invasive ventilation, and 9 had at least one central line during hospital stay (umbilical venous catheter: 3 patients; umbilical venous and epicutaneo-caval catheters: 3 patients; umbilical venous and arterial catheters and epicutaneo-caval catheter: 1 patient). Three patients had a central catheter at the onset of the colonization, and three patients had no central catheterization during their hospital stay. Parental nutrition was maintained for a median of 12 days (minimum 5 days; maximum 37 days). Nasogastric tube was present in all newborns for a mean time of 36.4 days (standard deviation 18.8 days). Trophic nutrition had a median introduction of two days of life (minimum 1 day; maximum 6 days). A median of 13 days was necessary to achieve exclusive enteral nutrition (minimum 4 days; maximum 37 days). Four neonates were being fed with both parenteral and enteral nutrition on the day of CPE isolation, and six babies had achieved full enteral nutrition by that time. Two newborns had pancytopenia, one had bicytopenia (anemia and neutropenia), and three did not show any cytopenia previously to the colonization. Of the five anemic patients, three received red blood cell transfusions. The mean length of stay was 50.4 days with a standard deviation of 28 days. The median days to patient colonization detection was 12.5 days (minimum 4 days; maximum 90 days).

In this study, two species of CPE (*K. pneumoniae:* 7 patients; *Enterobacter cloacae*: 3 patients) and three different types of carbapenemases (KPC: 4 patients; OXA-48: 2 patients; VIM: 4 patients) were identified. Most patients were colonized with KPC and one was co-colonized with extended-spectrum β-lactamase *Kluyvera ascorbata*. The triplets were all colonized with the same VIM *E. cloacae*. One newborn from a twin pregnancy was not colonized.

When the first CPE-colonized patient was identified, targeted prevention and infection control procedures were implemented for all newborns. Surveillance strategies included systematic microbiological screening of all NICU patients; contact isolation and cohorting of colonized newborns; improving healthcare environmental cleaning and disinfection; and both staff and parent education on hand hygiene and protective personal equipment usage. Staff cohorting was not possible.

None of the CPE-colonized newborns developed CPE infection. There was no associated mortality. Statistical significance was found in the bivariate analysis for birth weight, gestational age, length of stay, and days of central line as described in Table 1. No independent risk factors for CPE colonization were identified in the logistic regression analysis (X^2^ = 12.22, *p*-value ≤ 0.05), including the parameters with a *p*-value ≤ 0.05 in the univariate analysis (Table 2).

## 4. Discussion

Carbapenemase-producing Enterobacterales are an emerging group of pathogens in neonatal units; however, only a few studies have addressed this public health issue thus far [7,8]. CPE colonization risk factors, epidemiology, management, and outcomes have not yet been accurately described for this vulnerable population [1].

In this study, CPE gut colonization prevalence among neonatal patients was 5.8%, lower than some literature descriptions [1,4,10]. We were able to identify two Enterobacterales (*K. pneumoniae* and *E. cloacae*) with three different types of carbapenemases (KPC, OXA-48, and VIM), and KPC was found to be the most frequently isolated, as reported in the literature [2,8]. A median of 12.5 days to the first CPE isolation in rectal swabs suggests that hospital stay is an important risk factor for colonization and that screening procedures rationalization is possible without impairing the prompt identification of asymptomatic colonized newborns, as described in other studies [14].

The first identified colonized neonate was admitted from another intensive care unit in a different hospital, with adult, pediatric, and neonatal admissions for cardiac surgery. This unit was recently affected by a CPE colonization outbreak. Later, two newborns were found to be colonized by an OXA-48 producing *K. pneumoniae*. The VIM-producing *E. cloacae* was found in the triplets. As VIM is a less prevalent carbapenemase in our country [3,5], further investigation found that the triplets’ mother lived in India for the past year. Although we cannot establish a direct correlation, as mothers’ colonization status was not assessed, international traveling to CPE-endemic regions is described as a CPE colonization risk factor [1], raising concern about carbapenemase prevalence in the community [2]. In the future, it might also be useful to perform screening on the newborns’ mothers in order to identify more risk factors and clarify this complex issue.

Although CPE infection was a constant concern, no clinical infection or mortality was registered in our colonized newborns. In addition, although neutropenia is a known infection risk factor, its presence did not seem to influence infection development in asymptomatic colonized patients, as seen in other studies [6]. Severe infections with high morbidity and mortality and scarce antibiotic alternatives for neonatal patients are the most challenging features regarding CPE neonatal infection management [9]. The systematic approach used in our NICU enabled a prompt recognition of colonized patients and the introduction of effective infection control measures. We believe that in our sample, the continuous surveillance and cohorting of CPE-colonized neonates prevented further cross-transmission and progression to infection as described previously [1,7]. Moreover, the decreasing number of cross-colonized patients during this study probably reflects the healthcare workers’ infection control measures compliance [4]. Despite ideal recommendations [11], staff cohorting was not possible in our NICU due to limited human resources. Cross-transmission seemed to be the most probable form of colonization in our NICU, also as reported by other authors [6]. However, accurate transmission pathway identification was limited because the parents and the NICU’s healthcare staff were not screened. It should be noted that, in one set of twins, one of the babies was colonized with a VIM-producing *K. pneumoniae* while the other was not colonized during the hospital stay. This emphasizes the crucial importance and effectiveness of prompt isolation measures after the identification of colonized babies [4,12,16].

Regarding the colonized population, all mothers were hospitalized at least 48 h before delivery, and all newborns were exposed to invasive medical procedures and had a prolonged length of stay. It can be said that a higher need for invasive techniques and manipulation and longer hospitalization periods could have led to an increased risk of CPE colonization [11].

Trophic nutrition and progression to exclusive enteral feeding did not seem to be delayed by CPE colonization, and these were thought to be protective regarding nosocomial infections [11]. When comparing CPE-colonized and non-colonized patients, a statistically significant difference was found between medians of birth weight, gestational age, length of stay, and days of central line (bivariate analysis, Table 1). In this study, there were no independent risk factors for CPE colonization, which may be explained by its limitations, such as a small sample. Efforts to identify risk factors and colonization sources are crucial. We emphasize the importance of routine screenings of mothers and babies, especially those with prolonged hospital stays, and NICU staff and surfaces, such as incubators, counters, sinks, medical devices, floor, and walls [4,7]. In our NICU, parents have an active role in newborns’ care alongside the staff. Since carbapenemase prevalence is increasing in the community [2], weekly screening of caregivers could also be useful. The lack of these data are also limitations of the study.

In the literature, data regarding CPE colonization in an NICU’s critically ill patients is limited, and no Portuguese neonatal CPE colonization study has yet been published. Therefore, despite our small sample, this study provides important epidemiologic information and infection control strategies in this specific population. Colonized newborns can be a reservoir for transmission, and so early detection and infection control measures can decrease the risk of infection.

In our NICU, existing practices were strengthened, such as universal screening at admission and then on a weekly basis, hand hygiene education, the use of disposable gown and gloves while contacting with colonized newborns, protective regular review of invasive devices, adequate bed spacing, proper environmental cleaning, and segregation of waste, prioritizing patient contact isolation since admission, and cohorting, as proposed by other groups [4,11,12,13]. Although this approach demands significant human, financial, and laboratory resources, knowing CPE colonization rates in the NICU is crucial to guide empirical therapeutic options when Gram-negative infections are suspected [4,7,14].

## 5. Conclusions

An NICU’s CPE colonization or infection cases are a real threat to newborns worldwide. CPE colonization risk factors are still to be determined accurately in the neonatal population. Surveillance is the cornerstone of a good infection control program. A systematic approach is key to successfully control CPE and the associated complications.

## Figures and Tables

**Table 1:** Bivariate analysis of risk factors among carbapenemase producing Enterobacterales (CPE) at an neonatal intensive care unit (NICU).

	CPE-Positive N = 10	CPE-Negative N = 163	*p*-Value
Birth weight (mean in g, standard deviation)	1620.2 (867.2)	2477.7 (1007.1)	0.01
Gestational age (mean in days, standard deviation)	31.4 (3.5)	35.29 (4.6)	0.009
Length of stay (median in days, minimum; maximum)	45 (8; 99)	7 (1; 137)	<0.001
Central line (median in days, minimum; maximum)	9.5 (0; 47)	0 (0; 37)	0.008

**Table 2:** Multivariable analysis of risk factors for carbapenemase producing Enterobacterales (CPE) gut colonization.

	CPE-Positive N = 10	CPE-Negative N = 163	*p*-Value	Odds Ratio (95% CI)
Birth weight (mean in g, standard deviation)	1620.2 (867.2)	2477.7 (1007.1)	0.277	0.99 (0.99–1.01)
Gestational age (mean in days, standard deviation)	31.4 (3.5)	35.29 (4.6)	0.333	1.21 (0.83–1.76)
Length of stay (median in days, minimum; maximum)	45 (8; 99)	7 (1; 137)	0.053	1.03 (1.00–1.06)
Central line (median in days, minimum; maximum)	9.5 (0; 47)	0 (0; 37)	0.891	1.01 (0.92–1.10)

## Data Availability

The data presented in this study is contained within the article and available on request from the corresponding author.
